# Cepheid 9-gene host mRNA expression across the spectrum of *Mycobacterium tuberculosis* infection and disease: insights across the lifespan

**DOI:** 10.1016/j.ebiom.2026.106462

**Published:** 2026-09-01

**Authors:** Marva Seifert, Michael Gracia, Donald G. Catanzaro, Faiqa Rashid, Rehan R. Syed, Faisal Masood, Naomi Hillery, Alamdar Hussain, Ashley Swing, Rebecca E. Colman, Uzma Majeed, Antonino Catanzaro, Sabira Tahseen, Timothy C. Rodwell

**Affiliations:** a Division of Pulmonary, Critical Care, and Sleep Medicine, Department of Medicine, University of California San Diego, 9500 Gilman Drive, La Jolla, CA, 92093, United States; b Department of Pediatrics, University of California San Diego, 9500 Gilman Drive, La Jolla, CA, 92093, United States; c Department of Biological Sciences, University of Arkansas, 601 Science Engineering Hall, Fayetteville, AR, 72701, United States; d National Tuberculosis Control Program, National TB Reference Laboratory, CMU Offices, Block E and F, EPI Building, NIH Complex, Chak Shahzad, Islamabad, 44000, Pakistan; e Division of Infectious Diseases and Global Public Health, Department of Medicine, University of California San Diego, 9500 Gilman Drive, La Jolla, CA, 92093, United States

**Keywords:** Tuberculosis infection, Active tuberculosis disease, Diagnosis, Host-response, Adolescents and young adults

## Abstract

**Background:**

Novel diagnostic solutions using host-response-based gene signatures to differentiate between individuals infected with *Mycobacterium tuberculosis* presenting with active tuberculosis disease (ATB), individuals infected with the bacterium in the absence of active tuberculosis disease (TBI), and individuals with no evidence of ATB or TBI (no TB) have demonstrated limited utility. However, the impact of age-related immunological changes on diagnostic performance remains unclear.

**Methods:**

Using blood samples collected from 119 ‘adolescent/young adults’ aged 10–24 years, 259 ‘adults’ aged 25–55 years, and 79 ‘older adults’ aged 56 years and above we explored differences in expression of eight gene targets using a novel research-use-only Cepheid Xpert TB/LTBI assay. We plotted relative expression for each gene by age stratified by TB status, then constructed two composite classifiers using a subset of gene targets. These composite classifiers were compared using AUCs derived from logistic regression models fitted within each age stratum.

**Findings:**

Expression of *IL2*, *IFNG*, and *MIG* appeared to best differentiate individuals who were classified as TBI or ATB from no TB and were used to construct an ‘exposure’ classifier which yielded an AUC of 0.89 (95% CI 0.83–0.96) among adolescent/young adults, 0.82 (95% CI 0.76–0.87) among adults, and 0.71 (95% CI 0.58–0.84) among older adults. Similarly, expression of *SLPI*, *VEGFA*, *PLAU,* and *GBP5* appeared to best differentiate between individuals who were classified as ATB from TBI or no TB. The corresponding composite ‘disease’ classifier yielded an AUC of 0.88 (95% CI 0.80–0.96) among adolescent/young adults, 0.74 (95% CI 0.66–0.82) among adults, and 0.56 (95% CI 0.40–0.73) among older adults.

**Interpretation:**

This study demonstrates the importance of considering age in the development and evaluation of host-response-based diagnostics for ATB and TBI and suggests that host-response-based diagnostics may prove most useful in differentiation of ATB, TBI, and no TB among adolescent/young adults.

**Fundings:**

This study was supported by the US Department of Defence (award number W81XWH-18-1-0253) and National Institutes of Health (award number R01AI137681). Salary support was received from the National Institutes of Health (training grant number T32AI007384).

**Research in context**

**Evidence before this study**

Accurate detection of both *Mycobacterium tuberculosis* infection and active tuberculosis disease is critical for reducing ongoing transmission and improving treatment outcomes. While no universal, highly sensitive diagnostic for identifying both infection and active disease currently exists, novel host-response-based diagnostic solutions offer potential solutions. We searched PubMed for articles published from database inception to May 1, 2026, with no language restrictions using the search terms “tuberculosis infection or disease”, “host response diagnosis or diagnostic”, and “age or lifespan” to identify studies that assess the impact of age on the performance of host-response-based diagnostics. Although studies evaluating tuberculosis host-response-based diagnostic solutions do exist and several acknowledge the unique challenges of detecting tuberculosis infection and determining risk of progression to active disease among adolescents and young adults, few have evaluated the performance of these host-response-based diagnostic solutions in this age group.

**Added value of this study**

Our evaluation of the research-use-only Xpert TB/LTBI assay (Cepheid, USA) to identify both individuals with tuberculosis infection and active disease was conducted in Pakistan in 2022 and 2023. We observed significant differences in assay performance between adolescents and young adults, adults, and older adults. The assay was most effective in identifying both tuberculosis infection and active disease among adolescents and young adults, corresponding with the current understanding of immune system development and maturation.

**Implications of all the available evidence**

Our age-stratified analysis showed significant differences in transcriptomic signatures across the lifespan, demonstrating the importance of considering immune system response capacity and potential global exposures when developing and evaluating host-response-based diagnostic solutions for tuberculosis infection and active disease.

## Introduction

Approximately one quarter of the global population is infected with *Mycobacterium tuberculosis* (TBI), and an estimated 10.8 million of these individuals develop active tuberculosis disease (ATB) annually.^1^^,^^2^ Accurately identifying both individuals who are TBI and who have ATB is key to reducing ATB related morbidity and mortality. However, despite this critical need, commercially available diagnostic solutions that detect both TBI and ATB remain elusive.

A majority of diagnostics for ATB are pathogen-based, relying on the detection of *M. tuberculosis* using either culture, smear, or molecular based approaches, and are often complemented by radiological techniques to identify specific patterns of infection or disease.^3^ The sensitivity of both microbiological and radiological methods of ATB detection are imperfect and do not always identify all individuals who are likely to respond to treatment.^2^ Current host-response diagnostic tools measure antigen or mitogen stimulated immune responses among individuals either *in vivo*, as with tuberculin skin testing (TST), or *in vitro*, in the case of interferon-γ release assays (IGRAs), and should detect all individuals who are or have been infected with *M. tuberculosis* regardless of symptom or treatment status. However, these methods have been shown to miss up to 19% of infections among individuals with culture positive disease, indicating a need for improvement.^3^^,^^4^ Currently no reliable, blood-based diagnostic solutions exist that are able to discriminate between individuals with active ATB, individuals who are TBI, and individuals who are not infected nor have active disease (no TB).

Adding to the complexity of identifying individuals with ATB or TBI is the variation in incidence by age and immune function.^5^ ATB detection among children is notoriously difficult and is reflected in the lower sensitivities of current diagnostic solutions in this group.^6^ Differences in TBI detection by age are also apparent, as false negativity for TBI testing is highest among older aged individuals, and results are considered unreliable among infants and young children, suggesting specific markers of immune response that may be indicative of disease among adolescents and young adults may be less useful in young children and older adults.^7^^,^^8^

Novel approaches to ATB and TBI detection, including blood-based host gene expression signatures, have recently been explored with limited success.^9^, ^10^, ^11^ These signatures indicate that transcriptional states of circulating blood cells are significantly altered by the presence of *M. tuberculosis* antigens. While the risk of developing active ATB is understood to be highly correlated with host immune status, age, and age-related immune function, analysis by age has typically not been central when evaluating novel host response-based diagnostic tools. The current ‘one size fits all’ approach to host response-based detection methods is inconsistent with our current understanding of immune development across the lifespan and has likely contributed to their lower sensitivities.

To address these gaps, we evaluated a research use only (RUO) Xpert TB/LTBI assay (Cepheid, USA), which measures levels of host mRNA after stimulation with *M. tuberculosis* antigens ESAT6 and CFP10. Eight biomarkers (*GBP5, VEGFA, MIG, IFNG, IL2, GBP1P1, PLAU,* and *SLPI)* associated with ATB, classic immune stimulation, and disease processes and a housekeeping gene (*CDE3)* are included in the assay.^10^^,^^12^^,^^13^ *GBP5* had been evaluated in the *M. tuberculosis* Host Response cartridge (Cepheid, USA) previously.^14^ Thus, the TB/LTBI assay combined two approaches—one for TBI detection and one for ATB detection—to differentiate between individuals with ATB, TBI, and no TB. We hypothesised that age-stratified analysis would lead not only to an improved understanding of host immune response to *M. tuberculosis* infection and disease but could also influence future evaluation of host response-based diagnostic solutions.

## Methods

### Study design

A prospective exploratory diagnostic study was conducted in partnership with the National TB Reference Laboratory of Pakistan at the Federal General Hospital in Islamabad and the Rawalpindi Leprosy Hospital in Rawalpindi between May 2022 and May 2023.^14^ The total number of samples collected was based on a sample size estimate powered to detect a difference between the index test and the updated WHO framework for evaluation of new tests for tuberculosis infection sensitivity and specificity targets.^15^ In brief, sequential patients aged five and above presenting with symptoms consistent with TB, who were referred for Xpert MTB/RIF Ultra (Cepheid, USA) testing by their treating clinician, and who had been entered into their respective hospital's presumptive ATB register, were eligible for the study. Individuals were excluded if they had initiated treatment for ATB any time during the previous six months, were incarcerated, or were institutionalised. After consenting, participants were enrolled, interviewed by study personnel, asked to provide clinical samples, and had data extracted from their medical records. Participants who were not diagnosed with ATB (either by Xpert MTB/RIF Ultra, liquid culture, or clinician diagnosis) were contacted three months post enrolment.

### Ethics

The study was conducted in accordance with the principles of the Declaration of Helsinki. The protocol was approved by the institutional review board of University of California San Diego (804856), the Office of Human Research Oversight at the US Department of Defence (E00212.1g), and the National Bioethics Committee of Pakistan (NBC-575). All participants provided written informed consent prior to enrolment in the study.

### Composite TB status classification

Participants with positive results on either liquid mycobacterium growth indicator tube (MGIT) culture (Becton Dickinson, USA), Xpert MTB/RIF Ultra (including trace results), or both were classified as having bacteriologically confirmed ATB. Participants who had radiological results consistent with ATB or were clinically diagnosed by their treating physician and who were placed on treatment were also classified as having ATB. Additionally, to ensure that all incipient cases of ATB disease were included, participants not diagnosed with ATB at enrolment were contacted for a three-month follow-up and, if at that time were diagnosed with ATB and placed on treatment were also classified as having ATB. Participants who returned a positive QuantiFERON-TB Gold plus (QFT-plus) (Qiagen, USA) result but were not diagnosed with ATB either at enrolment or during follow-up were classified as TBI. Participants who were classified as not having ATB, did not have radiological results indicative of ATB, and had a negative or indeterminate result on QFT-plus were determined to be ‘no TB’. For the overall discriminatory analysis TB status was further collapsed into two binary measures, TB infection status and TB disease status. TB infection differentiated individuals with TBI or ATB from individuals classified as no TB, and TB disease differentiated individuals with ATB from individuals with TBI or no TB.

### Cepheid Xpert TB/LTBI assay

Approximately 1 mL venous blood was collected in RUO Lionex tubes (KABE Primavette 1.4 mL Lithium heparin tubes containing recombinant ESAT-6 and CFP-10 antigens) from each participant and incubated for a minimum of 3 h at 37 °C. Partway through the study a protocol change resulted in a subset of samples being incubated overnight, increasing incubation times to up to 20 h. After incubation, a 400 μL aliquot of blood was then transferred into a vial containing Xpert Lysis Reagent. The TB/LTBI assays were designed to automate nucleic acid isolation, cDNA synthesis, and parallel qPCR. At the initiation of the study TB/LTBI assays were not available and sample aliquots were stored at −80 °C after the lysis step. Once TB/LTBI assays became available, stored samples were thawed at room temperature for 30 min to 2 h and 1 mL of blood/lysis specimen was transferred into the TB/LTBI assay for processing and analysis using the GeneXpert instrument, this was considered the ‘frozen’ workflow. After the TB/LTBI assays became available, samples were no longer frozen after the lysis step but instead were processed using a ‘live’ workflow. These samples followed the same protocol for collection, incubation, and lysis, but were directly transferred to the TB/LTBI assay for testing after the lysis step and did not undergo a freeze/thaw cycle. Both workflow and incubation procedures were recorded by blinded personnel for each sample. Cycle threshold (Ct) data were exported from the GeneXpert instrument and converted to CSV format.

### Statistics

Participants were divided into three age categories, ‘adolescent/young adults’ aged 10–24 years, ‘adults’ aged 25–55 years, and ‘older adults’ aged 56 years and above. Demographic and clinical factors were compared between age groups using appropriate statistical tests. Relative Ct values were calculated for each gene by subtracting the target gene Ct from a housekeeping gene Ct, *CDE3*, then dividing the result by the *CDE3* Ct value to obtain the relative difference of the target gene expressed when compared to the housekeeping gene. Gene-specific Ct values greater than or equal to 40 were treated as missing and excluded listwise from the analysis. Relative Ct values were presented visually for all gene targets assessed using a heatmap and stratified by age category and TB status. Additionally, scatter plots overlayed with fitted lines and 95% confidence intervals for TB status were used to visualise the distribution of relative Ct value for each gene target by age. To account for non-normality, the Wilcoxon rank-sum test was used to determine if there were any significant differences in relative Ct value distribution for each gene by process workflow (live vs frozen), incubation time (same day vs overnight incubation), diabetes status (self-reported disease vs no report), and sex (male vs female). Two composite classifier variables, the first constructed by summing the relative Ct values of *IL2*, *IFNG*, and *MIG* to differentiate between individuals classified as having ATB or TBI vs individuals classified as no TB and the second constructed by summing the relative Ct values of *SLPI*, *VEGFA*, *PLAU*, and *GBP5* to differentiate between individuals classified as having ATB vs individuals classified as TBI or no TB, were compared against two binary composite TB status variables, TB infection (TBI or ATB vs no TB) and TB disease (ATB vs TBI or no TB). Logistic regression models were constructed for each classifier to evaluate their ability to discriminate between the binary TB status variables while controlling for process workflow, incubation time, and diabetes status. These models were then used to determine AUCs for all study participants and for each age category. All analyses were conducted using STATA17 (StataCorp, USA). Data was not blinded for analysis.

### Role of funders

Funding agencies did not have a role in the study design, data collection, data analyses, interpretation, or writing of report.

## Results

**Fig. 1:**
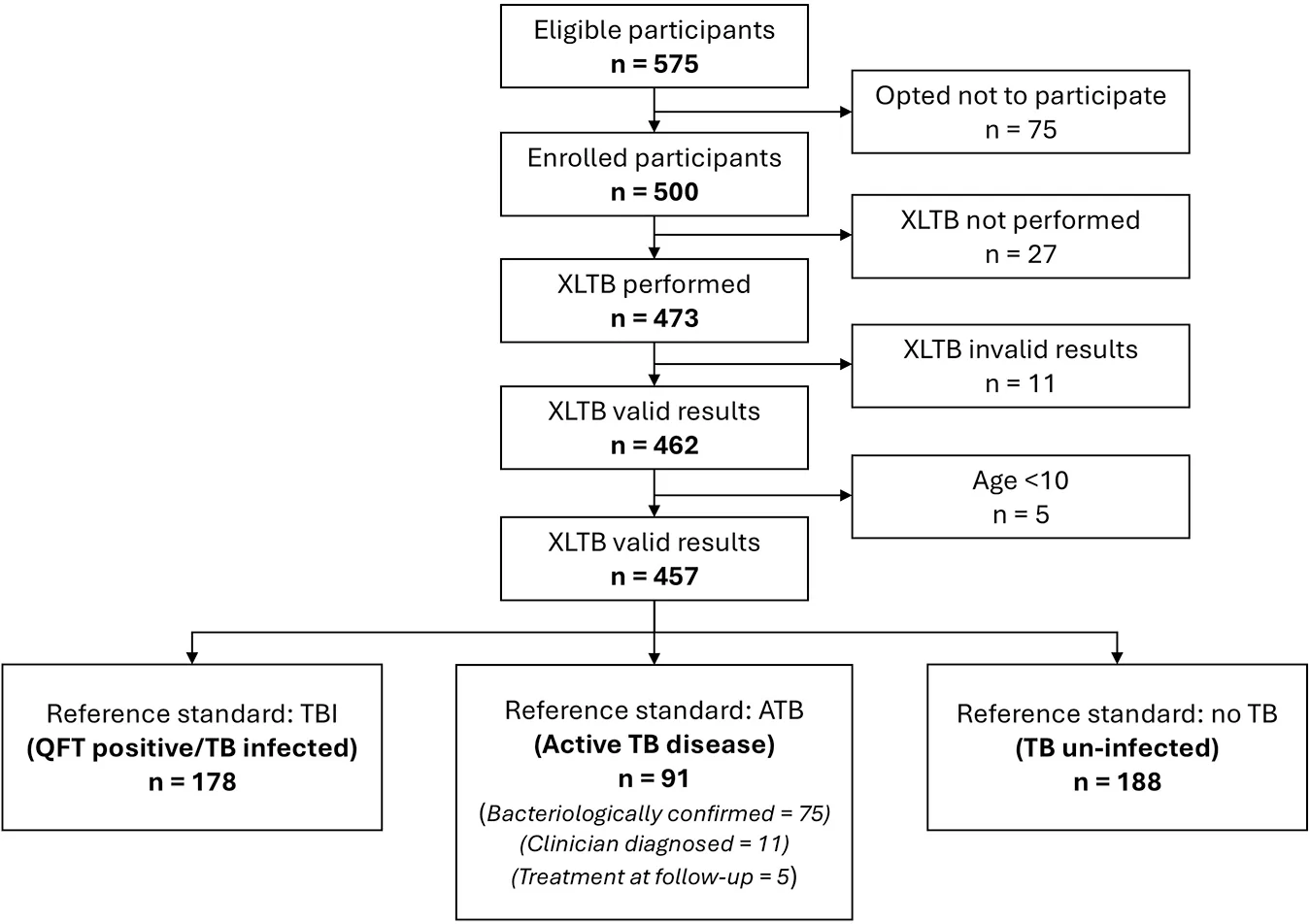
Participant enrolment flow chart.

The TB/LTBI assay was used to analyse venous blood samples from 473 participants, and valid results were returned for 462 (98%) of participants tested. Due to known differences in disease presentation prior to adolescence and our emergent understanding of differences in the immune response to *M. tuberculosis* infection and disease presentation over the life course, 5 participants less than age 10 were excluded from analysis, resulting in a total of 457 results for evaluation (Fig. 1).

### Participant characteristics

Demographic, diagnostic, and clinical characteristics were compared across age categories (Table 1). Mean age for adolescents/young adults, adults, and older adults was 18.8 (SD ± 3.8), 38.3 (SD ± 8.5), and 62.5 (SD ± 5.8) years, respectively. Individuals in the older adult age category were significantly more likely to have abnormal radiologic findings (p-value <0.001) compared to adolescent/young adults and adult age categories. Adults were significantly more likely to return a positive QFT-plus result (p-value 0.029) compared to both adolescent/young adults and older adults. There were no significant differences in reported symptoms or composite TB status across the age categories.

**Table 1:** Characteristics of study participants by age category.

	Adolescent/young adult	Adult	Older adult	Total
	119	259	79	457
Demographic characteristics, n (%)				
Male	60 (50.4)	139 (53.7)	43 (54.4)	242 (53.0)
Age, mean (SD)^a^	18.8 (3.8)	38.3 (8.5)	62.5 (5.8)	37.4 (15.8)
Clinical characteristics, n (%)				
HIV positive	2 (1.7)	2 (0.8)	1 (1.3)	5 (1.1)
Diabetes^b^	0	20 (7.7)	12 (15.2)	32 (7.0)
History of TB	15 (12.6)	57 (22.0)	23 (29.1)	95 (20.8)
Reported symptoms				
Night sweats	30 (25.2)	61 (23.6)	24 (30.4)	115 (25.2)
Persistent cough	98 (82.4)	218 (84.2)	71 (89.9)	387 (84.7)
Weight loss	85 (71.4)	162 (62.5)	56 (70.9)	303 (66.3)
Xray result^b^				
Normal	56 (47.1)	105 (40.5)	12 (15.2)	173 (37.9)
Abnormal	39 (32.8)	124 (47.9)	57 (72.2)	220 (48.1)
Not done	24 (20.2)	30 (11.6)	10 (12.7)	64 (14.0)
TB diagnostic results, n (%)				
QFT-plus result^b^				
Positive	52 (43.7)	151 (58.3)	39 (49.4)	242 (53.0)
Negative	52 (43.7)	75 (29.0)	25 (31.6)	152 (33.3)
Indeterminate	15 (12.6)	33 (12.7)	15 (19.0)	63 (13.8)
Smear positive	6 (5.0)	15 (5.8)	5 (6.3)	26 (5.7)
Culture positive	15 (12.6)	31 (12.0)	12 (15.2)	58 (12.7)
Xpert MTB/RIF Ultra positive	15 (12.6)	39 (15.1)	14 (17.7)	68 (14.9)
MTB Trace	0 (0)	9 (3.5)	3 (3.8)	12 (2.6)
MTB very low/low	10 (8.4)	16 (6.2)	6 (7.6)	32 (7.0)
MTB medium/high	5 (4.2)	14 (5.4)	5 (6.3)	24 (5.3)
Composite TB status, n (%)				
TB disease (ATB)	22 (18.5)	52 (20.1)	17 (21.5)	91 (19.9)
TB infected (TBI)	37 (31.1)	111 (42.9)	30 (38.0)	178 (38.9)
TB uninfected (no TB)	60 (50.4)	96 (37.1)	32 (40.5)	188 (41.1)

a ANOVA p-value<0.05.

b Chi square p-value <0.05.

### Gene specific relative Ct values by TB status

**Fig. 2:**
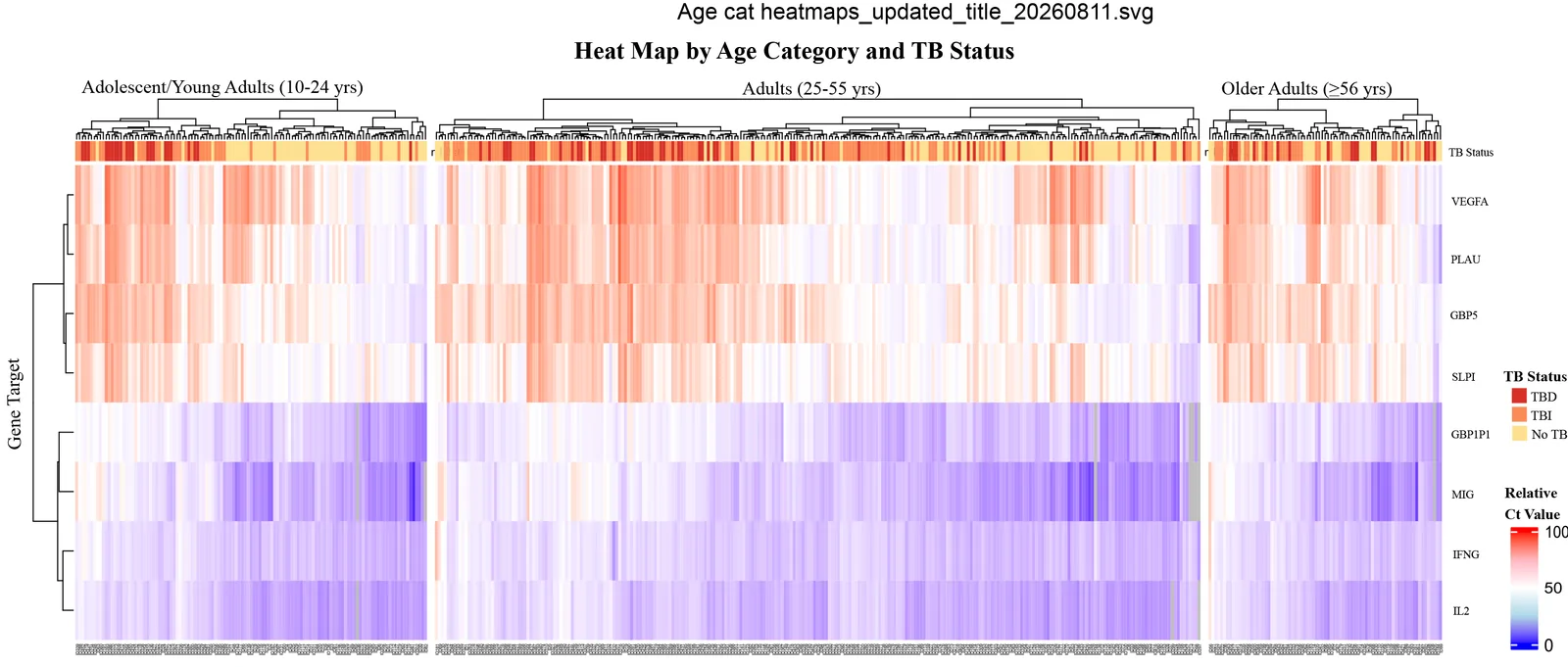
Heat map of relative Ct value for each gene target by age category with TB status.

**Fig. 3:**
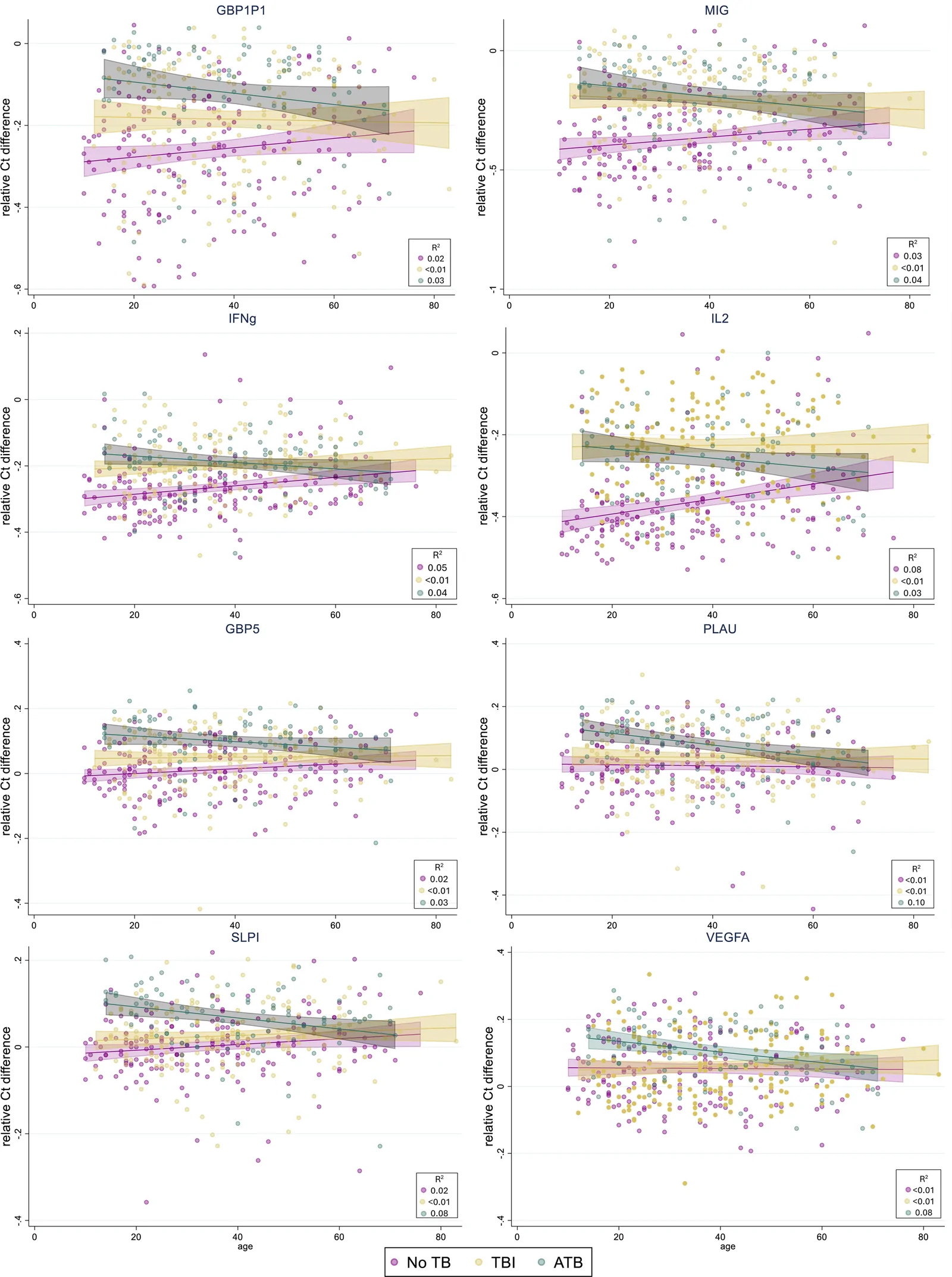
Distribution of gene expression by age stratified by TB status. For each gene assessed, relative Ct differences were plotted by age and overlayed by fitted TB status lines and 95% confidence intervals bands (green for individuals classified as having ATB, tan for TBI, and purple for no TB). As a measure of fit between relative Ct difference and age, R^2^ are included for each gene stratified by TB status.

Relative Ct values for each gene (rows) by TB status (columns) were stratified by age category (overarching columns) using a heat map (Fig. 2). Clustering by TB status was apparent for *SLPI*, *GBP5,* and *PLAU* which were downregulated among individuals with TBI and no TB and for *GBP1P1, IL2*, *MIG*, and *IFNG*, *VEGFA* which appeared globally upregulated among individuals with ATB and TBI, however intensity of relative Ct values appeared to vary by age category. Relative Ct values were presented in Table 2 and plotted in Fig. 3. While there was considerable variation in individual relative Ct values, a consistent global trend was observed for *IL2*, *IFNG*, and *MIG*, where the distributions of relative Ct values were more similar for individuals classified as having ATB or TBI compared to those classified as no TB. In contrast, the distributions of *SLPI*, *VEGFA*, and *PLAU* relative Ct values were more similar for individuals classified as no TB or TBI compared to those with ATB. For *GBP1P1* and *GBP5*, the distributions of relative Ct values were higher for individuals classified as having ATB compared to those classified as TBI, and similarly the distribution of relative Ct values was higher for individuals classified as TBI compared to those classified as no TB. These visual differences were less pronounced as individuals aged and fitted lines appeared to converge for individuals aged 55 and above.

**Table 2:** Gene specific relative Ct values (mean and standard deviation) and classifier variable results by age category and TB status.

TB status	No TB			TBI			ATB		
Age in years	10–24	25–55	≥56	10–24	25–55	≥56	10–24	25–55	≥56
*VEGFA*	0.056 (0.091)	0.047 (0.105)	0.071 (0.095)	0.056 (0.102)	0.056 (0.101)	0.08 (0.095)	0.140 (0.077)	0.111 (0.080)	0.038 (0.093)
*PLAU*	0.001 (0.087)	0.011 (0.100)	0.021 (0.123)	0.043 (0.097)	0.036 (0.105)	0.042 (0.08)	0.121 (0.070)	0.082 (0.085)	0.022 (0.113)
*GBP5*	0.000 (0.072)	0.008 (0.078)	0.042 (0.075)	0.052 (0.087)	0.046 (0.091)	0.060 (0.061)	0.131 (0.077)	0.095 (0.083)	0.073 (0.093)
*SLPI*	−0.011 (0.068)	0.004 (0.072)	0.024 (0.094)	0.027 (0.053)	0.024 (0.080)	0.032 (0.068)	0.114 (0.044)	0.057 (0.070)	0.046 (0.081)
*GBP1P1*	−0.275 (0.141)	−0.267 (0.134)	−0.211 (0.143)	−0.187 (0.164)	−0.186 (0.132)	−0.178 (0.121)	−0.066 (0.103)	−0.130 (0.132)	−0.158 (0.110)
*MIG*	−0.397 (0.163)	−0.369 (0.167)	−0.328 (0.198)	−0.191 (0.190)	−0.213 (0.169)	−0.230 (0.175)	−0.133 (0.173)	−0.192 (0.177)	−0.256 (0.177)
*IFNG*	−0.281 (0.079)	−0.270 (0.092)	−0.224 (0.097)	−0.202 (0.098)	−0.200 (0.085)	−0.182 (0.063)	−0.149 (0.085)	−0.198 (0.082)	−0.211 (0.065)
*IL2*	−0.397 (0.080)	−0.362 (0.107)	−0.318 (0.137)	−0.233 (0.116)	−0.220 (0.102)	−0.241 (0.107)	−0.206 (0.077)	−0.265 (0.098)	−0.291 (0.121)
‘Disease’ classifier	0.053 (0.260)	0.071 (0.292)	0.158 (0.331)	0.178 (0.294)	0.175 (0.305)	0.214 (0.245)	0.506 (0.219)	0.345 (0.252)	0.178 (0.342)
‘Exposure’ classifier	−1.063 (0.264)	−1.000 (0.329)	−0.870 (0.401)	−0.624 (0.386)	−0.626 (0.319)	−0.653 (0.310)	−0.436 (0.195)	−0.643 (0.293)	−0.756 (0.334)

### Impact of process workflow, incubation time, diabetes status, and sex on relative Ct values

Relative Ct value distributions were significantly different (p-value <0.05) between ‘live’ and ‘frozen’ process workflows for each gene assessed in at least one TB status category (data not shown). Additionally, significant differences (p-values <0.05) in the distribution of relative Ct values by incubation time were identified in at least one TB status category for all genes except for *IFNG* and by diabetes status for *SLPI*. There were no significant differences (p-values >0.05) in distribution of relative Ct values by sex. Based on these findings, process workflow, incubation time, and diabetes status were included in AUC analysis of classifier variables.

### Composite classifier variables

Given the differential distribution of relative Ct value by gene cluster, two composite classifier variables were constructed and evaluated as potential diagnostic biomarkers. First, an ‘exposure’ classifier variable to identify all individuals exposed to and infected with *M. tuberculosis*, individuals with TBI or ATB, from individuals classified as no TB but presenting with ATB-like symptoms, was constructed by summing the relative Ct values of *IL2*, *IFNG*, and *MIG.* While *GBP1P1* appeared to distinguish between all three TB statuses its inclusion in the exposure classifier did not increase the AUC and was therefore excluded from the classifier. There was a marked difference in distribution of the exposure classifier among younger individuals, however, as age increased the fitted lines for TB status converged reducing the potential utility of this exposure classifier as a diagnostic biomarker to diagnose TB in older adults. Overall, the AUC for the exposure classifier controlling for both incubation period and process workflow was 0.823 (95% CI 0.780–0.865). Specific age category AUCs are presented in Table 3. Among adolescent/young adults the AUC for the exposure classifier was 0.897 (95% CI 0.833–0.960), and among adults and older adults the AUCs were 0.814 (95% CI 0.754–0.872), and 0.731 (95% CI 0.610–0.852), respectively.

**Table 3:** Area Under ROC Curves (AUC) and 95% asymptotic normal confidence intervals of logistic regression models for constructed ‘exposure’ and ‘disease’ classifiers by overall study population and age stratum.

	Total N	AUC (95% CI)
**Exposure classifier** (detection of individuals with either TBI or ATB)		
Study population	443	0.823 (0.780, 0.865)
Ages 10–24	114	0.891 (0.826, 0.956)
Ages 25–55	251	0.816 (0.758, 0.874)
Ages 56 and above	78	0.710 (0.582, 0.838)
**Disease classifier** (detection of individuals with ATB)		
Study population	456	0.743 (0.684, 0.801)
Ages 10–24	119	0.880 (0.800, 0.959)
Ages 25–55	258	0.740 (0.663, 0.817)
Ages 56 and above	79	0.564 (0.402, 0.725)

A second composite ‘disease’ classifier variable was constructed to identify individuals with ATB from those classified as TBI or no TB. This variable was constructed by summing the relative Ct values for *SLPI*, *VEGFA*, *PLAU,* and *GBP5*. Similar to the exposure classifier, there was a marked difference in the distribution of the disease classifier values by TB status among younger individuals. As age increased, however, the fitted lines again converged. The AUC of the disease classifier controlling for both incubation period and process workflow was 0.734 (95% CI 0.675–0.792) overall. Specific age category AUCs were are presented in Table 3, and among adolescent/young adults the AUC was 0.881 (95% CI 0.802–0.960), and among adults and older adults the AUCs were 0.728 (95% CI 0.652–0.804) and 0.783 (95% CI 0.649–0.916), respectively.

## Discussion

Overall, the increase in relative Ct value of *IFNG, IL2,* and *MIG* expression in participants who are either classified as having ATB or TBI compared to individuals classified as no TB is consistent with the diagnostic principle underlaying commercial IGRAs which quantify the IFN-γ protein secreted by T cells when exposed to the same *M. tuberculosis* antigens used in this study (ESAT-6 and CFP-10). *IL2* and *MIG*, genetic correlates of *IFNG,* code for central components of the immune response, the former a vital stimulating cytokine for the growth and differentiation of helper, cytotoxic, and regulatory T cells and the latter (also known as CXCL9) a regulator of immune cell migration, differentiation, and activation which correlate with immune response to *M. tuberculosis* infection.^16^^,^^17^

The increase in relative Ct values for *PLAU*, *SLPI*, and *VEGFA* observed among participants classified as having ATB but not among those classified as TBI or no TB also aligns with gene function. *PLAU* encodes the protein urokinase which is involved ultimately in thrombolysis or extracellular matrix degradation^18^ and *SLPI* protects against proteolytic activation and degradation by proteases.^19^ *VEGFA* encodes a protein which acts on endothelial cells increasing vascular permeability, inducing angiogenesis, and promoting cell migration and has been previously identified as a biomarker for TB migration.^20^^,^^21^ It is reasonable then to speculate that genes which encode proteins essential to thrombolysis, tissue invasion, vascular permeability, T cell extravasation, and vascular formation would be upregulated in a state of tissue-invasive disease (ATB) vs controlled or latent infection (TBI).

Relative Ct values for *GBP5* and *GBP1P1* appeared to distinguish between all three TB statuses. *GBP5* and *GBP1P1* (also known as *GBP1*) and have been identified as markers of progression from TBI to ATB.^7^^,^^22^ The upregulation of *GBP5* has been shown to differentiate active ATB from healthy controls, TBI, and other diseases including acute sarcoidosis,^10^^,^^23^ however the non-specificity of its expression in other granulomatous diseases such as sarcoidosis, may act as a limiting factor for its use for TB diagnosis in older populations.^14^

Evaluation of fitted lines of relative Ct values for each gene by TB status and age revealed a few key trends, particularly among younger participants. The relative Ct values of *MIG*, *IL2*, and *IFNG* appeared to best distinguish participants classified as having ATB or TBI from those classified as no TB. Our constructed ‘exposure’ classifier (the sum of the relative Ct values for *MIG*, *IL2*, and *IFNG*) achieved an AUC of 0.823 for detection of participants with ATB or TBI vs participants classified as no TB for the entire study population. Among adolescents/young adults this classifier achieved an AUC of 0.897. While no current commercial diagnostic solutions are marketed for detecting and discriminating between ATB and TBI, IGRAs detect *M. tuberculosis* specific immune responses to both ATB and TBI and could be considered analogous to our constructed exposure classifier.

The ability to differentiate individuals with ATB from TBI using a host-response measure does not currently exist as a commercially available diagnostic, however several studies have evaluated possible candidates with some success.^24^^,^^25^ In our limited data set, the summative Ct values for *PLAU*, *VEGFA*, *SLPI*, and *GBP5* when plotted in aggregate as a constructed ‘disease’ classifier and were able to discriminate between those with ATB from TBI or no TB in the overall study population with an AUC of 0.734 and among adolescents/young adults an AUC of 0.881. Identification of *VEGFA* as a possible candidate for discrimination between ATB and TBI has been previously reported with an AUC of 0.8 and an AUC of 0.8 for GBP5, similar to our findings.^21^^,^^22^

Trends of relative Ct differences of *IL2*, *IFNG*, and *MIG* distinguishing individuals with TBI or ATB from those classified as no TB were most pronounced in the adolescent/young adult age category, possibly reflecting several unique aspects of the adolescent/young adult sub-population—specifically an opportune balance between the high degree of development of their adaptive (T cell) immune system and its lack of differentiation due to relatively low levels of global pathogen exposure and background inflammation. *IL2* is a central player in the proliferation of T lymphocytes and T cell response which are understood to increase through early adulthood.^26^ And, genes involved in their regulation may be up regulated during a period of exposure to or TBI if the immune system of an adolescent/young adult exists in such a hyper transcriptional and translational state. Furthermore, the breakdown or lack of differentiation between the expression of these three biomarkers in the older adult cohort suggests a high degree of both global exposures and of indiscreet background or baseline inflammation associated with atherosclerosis, dementia, and cancer in this age group. While poorly understood, this phenomenon is thought to add to the relative inefficacy of the elderly immune system, a concept sometimes referred to as senescence.^26^ As alluded to previously, these factors may render assays that measure immune response less discriminatory in this sub-population.

Despite these known differences in immunoregulatory function across the lifespan, most studies do not stratify results by age when assessing the utility of transcriptomic data for TB diagnosis, even though there is some evidence of IGRA utility for ATB diagnosis among children.^27^ Most studies have largely restricted study populations to only adults^23^^,^^28^^,^^29^ or children,^30^ or have included adults and children within their study population to identify a singular diagnostic signature without adjustment or stratification by age. While these studies do show similar age distributions between their cases and controls, only Olbrich et al. evaluated the moderating effect of age, although this study was conducted only with children under 15 years old.^30^ Even studies which provide the basis for the Cepheid *M. tuberculosis* Host Response prototype assay (MTB-HR) and treated age as a confounder showed no indication of testing its potential role as an effect modifier.^10^^,^^11^ It should perhaps not be surprising that a ‘one size fits all’ biomarker approach to development of host-response diagnostics leads to inconsistent and often unimpressive performance of many gene signatures^11^^,^^12^ and, after external validation, sensitivities and specificities fall below the WHO target product profile standards for diagnostic and triage tests.

Limitations of this study include the lack of external validation in diverse geographic enrolment sites, the use of a single dataset for classifier construction and evaluation, and the low prevalence of HIV in the study population. All participants were recruited from a single country and this potential lack of genetic heterogeneity may limit the cross-population generalisability of study findings.^31^ Using the same dataset to identify biomarkers for construction of discriminating classifiers and to evaluate the performance of these classifiers may introduce bias, however the use of an additive score likely mitigated this bias. Additionally, the low prevalence of HIV in the study population prevented any evaluation of the impact of HIV on host response within the study. Future studies among genetically diverse populations are warranted along with studies to clarify the specificity of these host-response profiles in the presence of other invasive or concurrent infections such as bacterial pneumonia and additional *M. tuberculosis* specific antigens.

In conclusion, it appears that the maximal response capacity of the adolescent/young adult immune system, combined with the relatively low degree of global exposures and background inflammation compared across the entire lifespan may create a ‘Goldilocks’ effect, demonstrating the potential utility of transcriptomic TB diagnostics within this age group. While the TB/LTBI assay may have significantly more utility among adolescent/young adults compared to older adults, further evaluation is needed to determine its generalisability. Finally, age effect must be considered when assessing and developing host response assays that quantify various aspects of the adaptive immune system response to *M. tuberculosis* infection and disease.

## Contributors

MS–conceptualisation, formal analysis, visualisation, writing original draft; MG–conceptualisation, writing review & editing; DGC–investigation, writing review & editing; FR–investigation; RRS–writing review & editing; FM–investigation, resources, supervision, data verification; NH–data curation, data verification, methodology, writing review & editing; AH–investigation, resources, supervision; AS–visualisation, formal analysis; REC–writing review & editing; UM–investigation; AC–funding acquisition; ST–methodology, project administration, investigation, resources, supervision; TCR–funding acquisition, supervision, writing review & editing. MS and MG contributed equally. All authors read and approved the final version of the manuscript.

## Data sharing statement

Data used in this study will be made available upon request from TCR (trodwell@ucsd.edu) after signed data access agreement with the University of California San Diego.

## Declaration of interests

AC and TCR are co-founders, board members and shareholders of Verus Diagnostics Inc, a company that was founded with the intent of developing TB diagnostic assays. AC and TCR have not received any financial support from Verus Diagnostics nor has Verus Diagnostics supported the development of the assay evaluated in this manuscript in any way, financial or otherwise. UCSD Conflict of Interest office has reviewed and approved their roles in Verus Diagnostics Inc. REC and TCR received salary support and travel cost reimbursement and MS received salary support according to their terms of a service contract between FIND and their home institution, the University of California San Diego. REC received funding for a grant to develop and evaluate a tNGS solution for drug resistance TB. MG, DG, RF, RS, FM, NH, AH, AS, UM, and ST have no conflicts to report.
